# Interfacial Modeling of Fibrinogen Adsorption onto LiNbO_3_ Single Crystal–Single Domain Surfaces

**DOI:** 10.3390/ijms22115946

**Published:** 2021-05-31

**Authors:** Jeffrey S. Cross, Yasuhiro Kubota, Abhijit Chatterjee, Samir Unni, Toshiyuki Ikoma, Motohiro Tagaya

**Affiliations:** 1 Department of Metallurgy and Ceramics Science, School of Science and Engineering, Tokyo Institute of Technology, 2-12-1 Ookayama, Meguro-ku, Tokyo 152-8552, Japan; kubota.35e.yasuhiro@jp.nssmc.com (Y.K.); srunni@gmail.com (S.U.); tikoma@ceram.titech.ac.jp (T.I.); 2 Department of Materials Science and Engineering, School of Materials and Chemical Technology, Tokyo Institute of Technology, 2-12-1 Ookayama, Meguro-ku, Tokyo 152-8552, Japan; 3 Dassault Systemes K.K., BIOVIA, ThinkPark Tower 21F, 2-1-1 Osaki, Shinagawa-ku, Tokyo 141-6020, Japan; 4 Department of Materials Science and Technology, Nagaoka University of Technology, 1603-1 Kamitomioka-cho, Nagaoka, Niigata 940-2188, Japan; tagaya@mst.nagaokaut.ac.jp

**Keywords:** LiNbO_3_, fibrinogen, adsorption, molecular dynamics, biosensor, XPS

## Abstract

For the development of next-generation protein-based biosensor surfaces, it is important to understand how functional proteins, such as fibrinogen (FBG), interact with polar substrate surfaces in order to prepare highly sensitive points of medical care diagnostics. FBG, which is a fibrous protein with an extracellular matrix, has both positively and negatively charged regions on its 3-dimensional surface, which makes interpreting how it effectively binds to polarized surfaces challenging. In this study, single-crystal LiNbO_3_ (LNO) substrates that have surface charges were used to investigate the adsorption of FBG protruding polar fragments on the positively and negatively charged LNO surfaces. We performed a combination of experiments and multi-scale molecular modeling to understand the binding of FBG in vacuum and water-solvated surfaces of LNO. XPS measurements showed that the FBG adsorption on LNO increased with increment in solution concentration on surfaces independent of charges. Multi-scale molecular modeling employing Quantum Mechanics, Monte Carlo, and Molecular Mechanics addressed the phenomenon of FBG fragment bonding on LNO surfaces. The binding simulation validated the experimental observation using zeta potential measurements which showed presence of solvated medium influenced the adsorption phenomenon due to the negative surface potential.

## 1. Introduction

LiNbO_3_ (LNO) is a widely used material for high-frequency surface acoustic wave (SAW) devices used in smartphones, etc., particularly for bandpass filtering due to its piezoelectric characteristic. SAW devices convert acoustic signals [1,2] into electromechanical signals and vice-versa. Since LNO is a piezoelectric material, it has ferroelectric bipolar domains, which exhibit an internal electric field, which also extends outside of its surfaces [3]. This electric field is known to influence the freezing point of liquids and other physical properties of materials in which they are in direct physical contact [4]. Because of these interesting combinations of properties, LNO has much potential for use as a biosensor either in optical or electronic applications [5,6]. To date, there have been a number of investigations using LNO for biosensing, but few have actually addressed the fundamental issues of surface adsorption characteristics, such as surface area of the adsorbent, temperature, pressure, activation of the solid adsorbent, and the type of adsorption (physisorption or chemisorption, etc.). Since the surface chemistry of LNO is unique in terms of its polarity (e.g., electric field), it is likely to enhance protein binding affinity. Fibrinogen (FBG) is a blood protein that is involved in blood clotting due to injury [7]. The physiological functions of FBG in the body in relation to normal plasma levels are described in detail in a newly published paper [8]. FBG is often employed as an adsorption model protein with a moderate molecular weight of 340 kDa [4,5,6,9,10]. Specifically, the FBG hexametric molecule has a rod-like shape with dimensions of 9 × 47.5 × 6 nm with a negative net charge at physiological pH (isoelectric point: pI = 5.8). The two end nodules (forming the C-terminal portions of the D regions) are similar and are made of the C-terminal ends of β and γ chains, while the center is a slightly smaller nodule (within the E region) that consists of the N-terminal ends of the six polypeptide chains. The central nodule is connected to the distal β-nodules and γ-nodules through two elongated coiled-coil regions [11].

For sensor applications, the adsorption of gas molecules on LNO was studied computationally, but there are few reported models explaining the protein adsorption at the liquid–solid interface. Recently, some studies have addressed the FBG adsorption onto various inorganic surfaces, such as titanium dioxide [11,12] and hydroxyapatite (HAp) [13,14]. For example, Monkawa et al. analyzed the FBG adlayer structures on HAp by quartz crystalline microbalance with dissipation technique and suggested the importance of evaluating the interfacial structures on the adsorption [13]. FBG adsorption on hydroxyapatite (HAp) with the FBG conformational changes of dumbbell-like structures has been described [14,15]. One of the αC domains, charged positive, is bonded to the negative sites of the HAp surface similar to phosphate and/or hydroxyl ions like in a “tail-on” bonding model, which changed from “side-on” adsorption at the initial stage.

Yoshioka et al. suggested the adsorption amount, as well as the conformational changes of Hap, depend on the protein surface charges (i.e., acidic or basic prote [16]. The interaction of FBG with gold surfaces using molecular dynamics simulation (MD) has shown that the gold surface has a capacity to induce FBG conformational changes, likely triggering an inflammation response in biological fluids [17]. Therefore, the objective of this study is to understand the surface adsorption of FBG on LNO single crystals, single-domain polar substrates through a combined approach of experimental analysis and simulation. We implemented multi-scale molecular modeling using Quantum Mechanics and Molecular Mechanics to simulate adsorption site preferences and the interactions of FBG over the LNO surface were studied using Monte Carlo simulation, Density Functional Theory (DFT), and molecular dynamics simulations were used to study the interaction both in the presence and in the absence of water molecules.

## 2. Results and Discussion

### 2.1. Experimental Results

**Figure 1:**
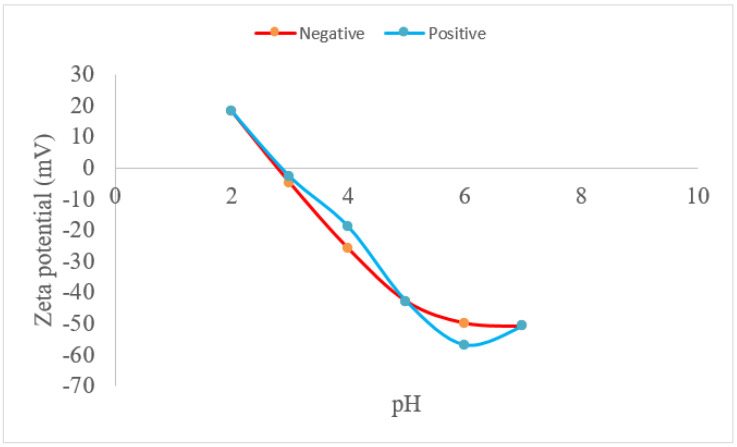
Zeta potential results of the single crystal-single domain LNO substrates with positive (001) and negative surfaces (00−1) (front and back side) after 10-min acid etching.

The zeta potential measurements of the etched LNO substrates showed the isoelectric point (pI) at 2.8, as shown in Figure 1. All the experiments with FBG solution adsorbed on LNO were carried out at a pH of 7.0, where the zeta potential was approximately −53 mV. It indicates that regardless of the surface polarity, the zeta potential of the LNO surface is unchanged. Previous zeta potential measurements of LNO nano-powder [18] were negative in the same range observed here, within experimental error. The electronegativity of the LNO surface closely resembled that of Nb_2_0_5_ [19]. Therefore, the polarity and the Li concentration on the surface of the LNO substrate appeared to have little effect on pH as observed from this method, but one can see that the negative surface had the least zeta potential at the same pH.

**Figure 2:**
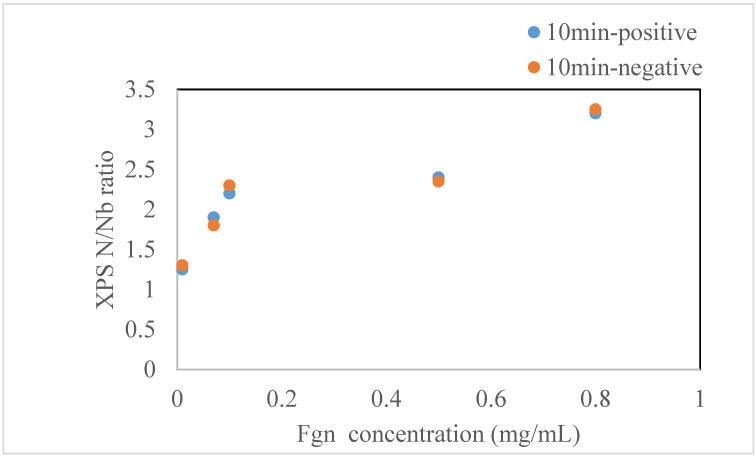
N/Nb ratio of LNO substrate by the XPS measurement as a function of the FBG concentration adsorbed on the surface.

As shown in Figure 2**,** FBG was adsorbed on the positive and negative surfaces of LNO. As confirmed from the XPS measurements, depending on the N/Nb ratio, there was little difference observed in the actual concentrations ranging from 0.01 to 0.5 mg/mL for 10 min. As the concentration of the FBG solution increased, the layer thickness of FBG adsorbed on the LiNbO_3_ surface increased, resulting in the Nb XPS peak decrease and higher N/Nb ratio.

### 2.2. Simulation Results and Discussion

We have performed a multi-scale simulation study with the protein fragments and a LNO supercell. This section consists of the following sub-headings, each with results from a specific approach. The sections consist of the following subheadings: (a) charge distribution calculation for the FBG protein structure, (b) facet area using a Monte Carlo method to find the best surface conducive to crystal growth, (c) the adsorption energy calculation of FBG fragment with a ceramic surface, (d) surface electrostatic potential and charge population calculation using Density Functional Theory (DFT) method, and (e) molecular dynamics calculation of the fragment and the surface in absence and presence of water.

#### 2.2.1. Charge Distribution Calculation for the FBG Protein Structure

**Figure 3:**
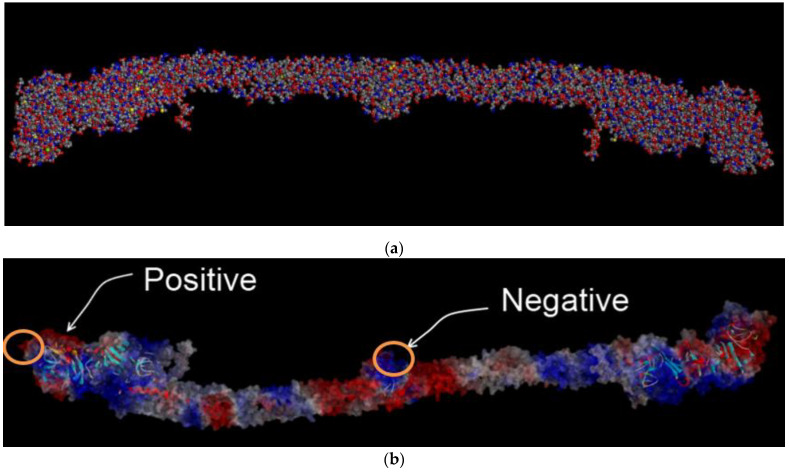
(**a**) Atomistic structure model of FBG. The color code for atoms are as follows: grey (carbon), blue (nitrogen), red (oxygen), yellow (sulfur). (**b**) FBG electrostatic potential map and protruding positive (red part) and negative (blue part) charged surface fragments used for surface simulation studies, which corresponds to the α*C* and *E* domains as described in the text.

FBG is a large and complex glycoprotein and shows a negative net charge at physiological pH (IP at pH 5.2) [20,21]. The net charge of the main body of fibrinogen present in the crystal structure is −8 (counting also the bound calcium ions). When including the flexible parts of the Aα-chains that are not visible in the crystal structure, the net charge becomes −12. We initially assumed the FBG, which has both positive and negative regions in Figure 3 on the surface, would perhaps bind at the different concentrations on the LNO substrate related to surface polarity; however, this was not the case in this study. We speculated that the near-surfaces of the LNO substrates were oxidized and react with H_2_O molecules to easily change the surface potential and its properties. Considering the hydration shell states on the substrate surfaces are an important factor influencing the protein adsorption [22,23,24], the experimentally measured adsorption differences between the positive and negative charges are extremely difficult to investigate. In order to understand the nature of the surface binding, an electrostatic map of the surface charge of the FBG molecule using discovery studio was prepared. The Delphi method used is an established method to predict protein interaction sites [21]. The map clearly indicated that electrostatic surface charge regions vary from positive (which is visualized as red) to negative regions (which are represented as blue). The regions, which had a neutral charge, are shown as gray regions. Figure 3a shows the structure model of FBG, and Figure 3b shows how the charges were distributed over twenty bins in the longitudinal direction of the FBG crystal structure. While the ends and the middle of the protein were net negatively charged, there were also parts of the protein that were net positively charged. Each FBG molecule was composed of two sets of alpha, beta, and gamma chains, which were joined together by disulfide bonds linked at cysteine residues. FBG consisted of 14 residues with sequence EGVNDNEEGFFSAR. Furthermore, FBG possessed a net negative charge at the end and middle part as well as contains net positively charged parts also. Previously, it had been shown that surface binding of FBG occurs at the end or the dumbbell region; however, it could also bind perpendicularly to the surface where the center region (negative) may contribute to binding. In FBG, the charge is distributed all over the protein, thus, we wanted to investigate the interaction between the charged surface of FBG with the LNO surface. Calculations were performed with the grouping of positive and negative charge separately to simplify the system, which might give direct evidence of interaction between the charged sites of FBG and ceramic surface. Based upon the electrostatic maps, we calculated the area of the charged regions. Then positive and negative fragments of the FBG were clipped carefully from the overall molecule by choosing a centroid of the charged region, making sure that amino acids remained intact. We used a fixed diameter of 22 Å. We then used the external-facing portion of these fragments to simulate the binding of these regions on both the positive and negative surfaces of LNO, as noted below.

We, therefore, truncated the smallest possible protein fragment to fit within the LNO surface supercell.

#### 2.2.2. Facet Area Using a Monte Carlo Method to Find the Best Surface Conducive to Crystal Growth

**Figure 4:**
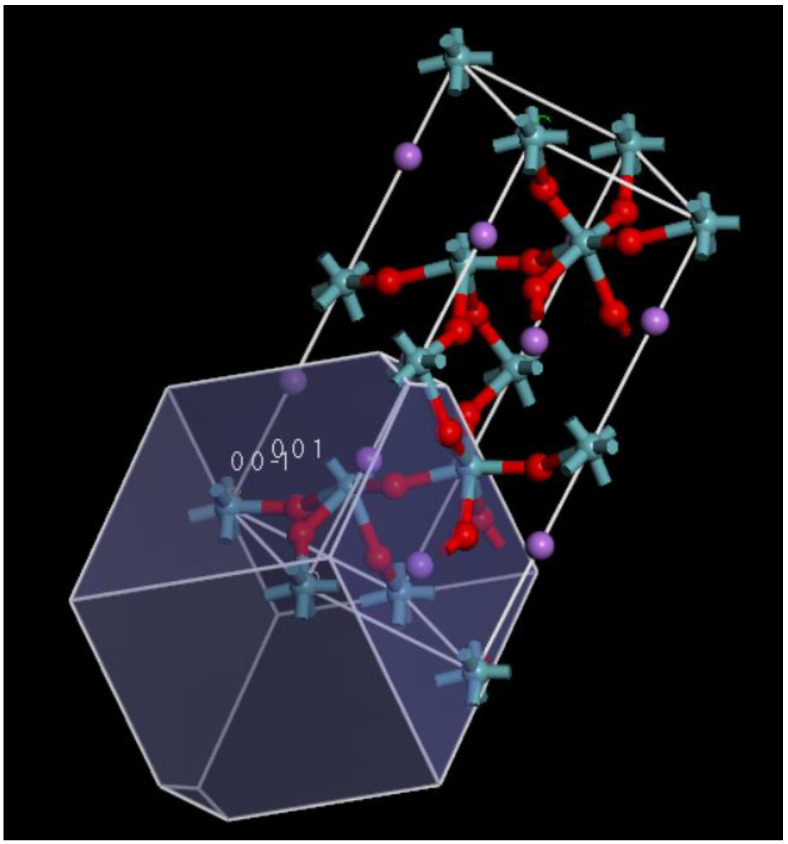
Morphology of LNO with possible surfaces; (001) and (00−1) labeled. The color code for atoms are as follows: cyan (niobium), pink (lithium), and red (oxygen).

We used the Monte Carlo method to correlate the surface area and the surface planes to propose the best surface conducive to the bulk growth of LNO. Figure 4 shows the unit cell with the most feasible surfaces. We showed the surface area of the possible surfaces in Table 1.

The results tabulated correlated to the plane distance in Å with a faceted surface area, indicating favorable growth directions. The results showed that the (001) and (00−1) surface was the plane with the highest facet area and was the growth plane. We, therefore, did all further calculations using the LNO (001) and the (00−1) surface. There were no experimental results available to verify the active surface. This type of method is used widely to measure the crystal facets to examine morphological preferences for binding [25].

#### 2.2.3. Calculated Adsorption Energy of FBG Fragment on LNO Surface

**Figure 5:**
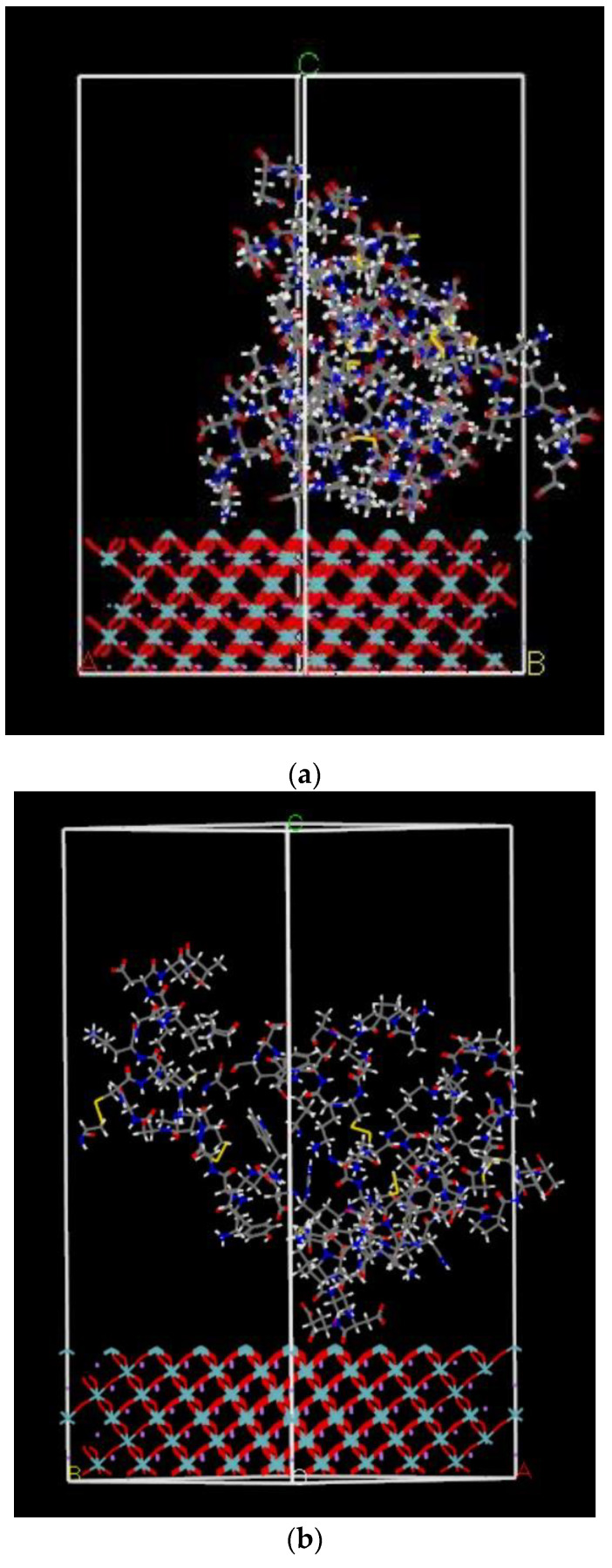
(**a**)The adsorption configuration of positive protein fragment over the (00−1) LNO surface. The color code is cyan (niobium), pink (lithium), red (oxygen), grey (carbon), blue (nitrogen), yellow (sulfur). (**b**) The adsorption configuration of negative protein fragment over (00−1) LNO surface. The color code for atoms are as follows: cyan (niobium), pink (lithium), red (oxygen), grey (carbon), blue (nitrogen), yellow (sulfur).

We then placed the protein fragment, either positive or negative fractions, onto the LNO surface to calculate the adsorption energy of the fragment and the surface. This is a Monte Carlo procedure to determine the most stable orientation of the fragment on the LNO surface. For simplification, adsorption was calculated assuming a vacuum slab above the 2 D surface; since adding water molecules would complicate the system and increase simulation time, the initial calculation was carried out in the gas phase. Both (001) and (00−1) surfaces were used in the simulation. The results are tabulated in Table 2 and Table 3 for two different surfaces with two different fragments. We show two representative figures with the results of adsorption energy calculation in Figure 5a,b. Since the fragment sizes of the positive and negative moiety of the protein fragments had different sizes and numbers of atoms, the adsorption energy values were also different. The positive fragment had 185 atoms, and the negative fragment had 175 atoms. The LNO surface was the same in size for both cases. An additional column in the table was added in terms of the adsorption energy per atom of each protein fragment to make a compatible comparison. This was similar to cohesive energy. The results revealed the affinity of positive and negative protein fragment for the (001) and (00−1) surface, respectively, as determined by the adsorption energy—the more negative the adsorption energy, the more stable the binding. To confirm whether the adsorption configuration obtained was the most appropriate one, we adopted Potential Mean Force (PMF) method using the Weighted Histogram Analysis Method (WHAM) [26] and the umbrella Integration Method [27,28,29]. We calculated the PMF for the center of the protein between the given beginning and end points. The path was divided into a number of steps. For each step, a MD simulation was run in which the molecular center was restrained to a fixed point on the path. The restraint acted as the umbrella potential. However, with the Monte Carlo calculations, we were unable to describe the appropriate scenario on the charge transportation, which is expected to be significant based upon the experimental adsorption results on the LNO surfaces. DFT calculations were performed and described to understand this in more detail in the next section.

#### 2.2.4. Surface Electrostatic Potential and Charge Calculation Using the DFT Method

**Figure 6:**
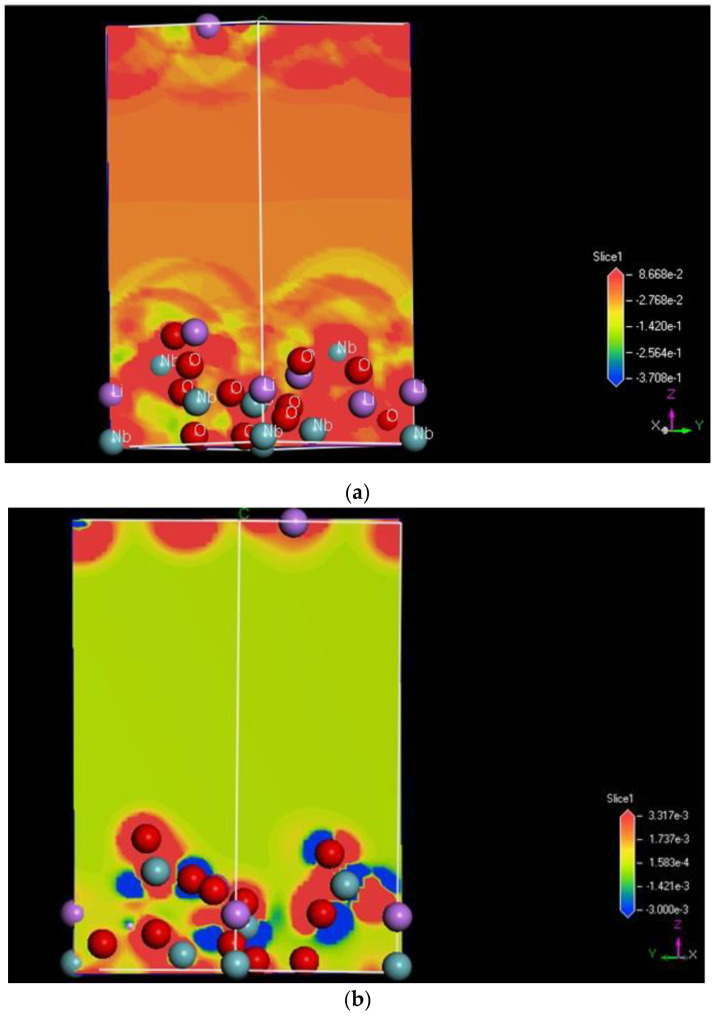

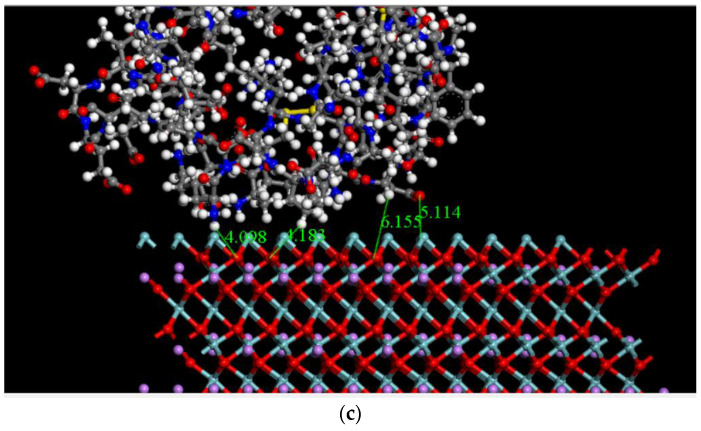
(**a**) Electrostatic potential map of the LNO Nb rich (00−1) surface. The color code for atoms are as follows: cyan (niobium), pink (lithium), red (oxygen). (**b**) Electrostatic potential with Nb poor LNO (001) surface. The color code for atoms are as follows: cyan (niobium), pink (lithium), red (oxygen). (**c**) The optimized structure of the positive protein fragment over the LNO (001) surface. The distance between the amino hydrogen to oxygen of LNO surface and the distance between the oxygen moieties to niobium is shown. The color code for atoms are as follows: grey (carbon), blue (nitrogen), yellow (sulfur), cyan (niobium), pink (lithium), red (oxygen).

To explain the adsorption behavior, one needs to calculate the change in the electrostatic potential and, consequently, the charge distribution to understand how the adsorption energy differences between two surfaces, such as one with more niobium on the LNO (00−1) and the other with less Niobium on the LNO (001) surface. To see the charge distribution over the unbound/free surfaces, we prepared a 2 × 2 × 1 surface for the LNO structure, comparing both (001) and (00−1) surfaces, and the results are shown in Figure 6 a,b, respectively. The electrostatic potentials were calculated over a range of −0.3 eV to 0.3 eV for both surfaces. We clearly observed that the surface charge was more positive for the Nb rich surface LNO (001) than for the LNO (001) surface with less Nb. The attached figures show the electrostatic potential from the best-fit plain. The color code is Red for positive and Blue for negative, and the color intensity is scaled with the potential spatial distribution. This was the reason we decided to carry out the binding energy simulation on the LNO (00−1) surface with a positive charge on the surface for preferential binding with the negative protein fragment and the LNO (001) surface with a more negative charge on the surface will bind the positive protein fragment.

We analyzed the binding energy of the protein fragments and charge transfer from the LNO surface by starting the calculation from the lowest energy structure obtained from the previous adsorption energy calculation. We then geometrically optimized the top layer of the surface and the whole protein fragment to calculate the binding energy of the fragment using the equation below. We observed that the protein fragment, which was separated from the LNO (001) surface, could come much closer to the surface, as shown on Figure 6c. A periodic boundary condition was imposed for all calculations. The remaining portion of the LNO atoms was fixed. The binding energy was defined as following: binding energy of protein fragment = Total energy of the complex (protein fragment and LNO surface)—(Total energy of the LNO surface + Total energy of the isolated protein fragment within the same periodic boundary condition]) which is a commonly used method to estimate binding energy.

The binding energy results, as shown in Table 4, indicating that the positive fragment bound more favorably to the (001) LNO surface, where the negative fragment was relatively unstable over (00−1) surface compared to the (00−1) surface.

The results showed varied binding energy justifying the hypothesis relating to the LNO surface having selective interactions with the fragments depending on the surface charge of the preferable protein. This methodology is a well-established rationale to explain the protein binding on metal surfaces [30]. Experimentally, it was hard to see this sort of dependency as the current experimental measurement in place was unable to capture the surface charge dependency on the binding of the protein. From the XPS, it was observed that the binding of protein increased with an increase in the concentration of the protein irrespective of the decrease in the Nb signal below the adsorbed FBG layer, as shown in Figure 2.

### 2.3. Dynamics Calculation Is Performed Using Molecular Mechanics Method with Water Molecules

**Figure 7:**
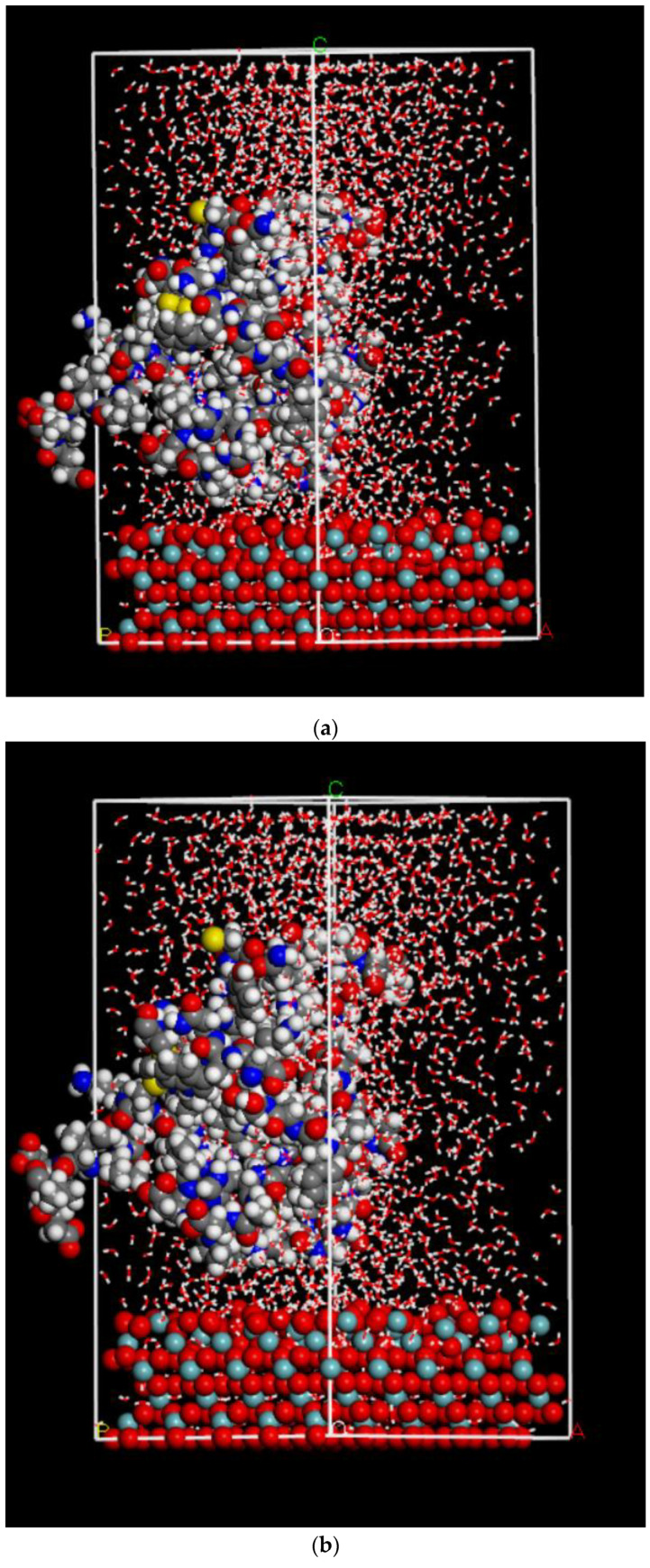

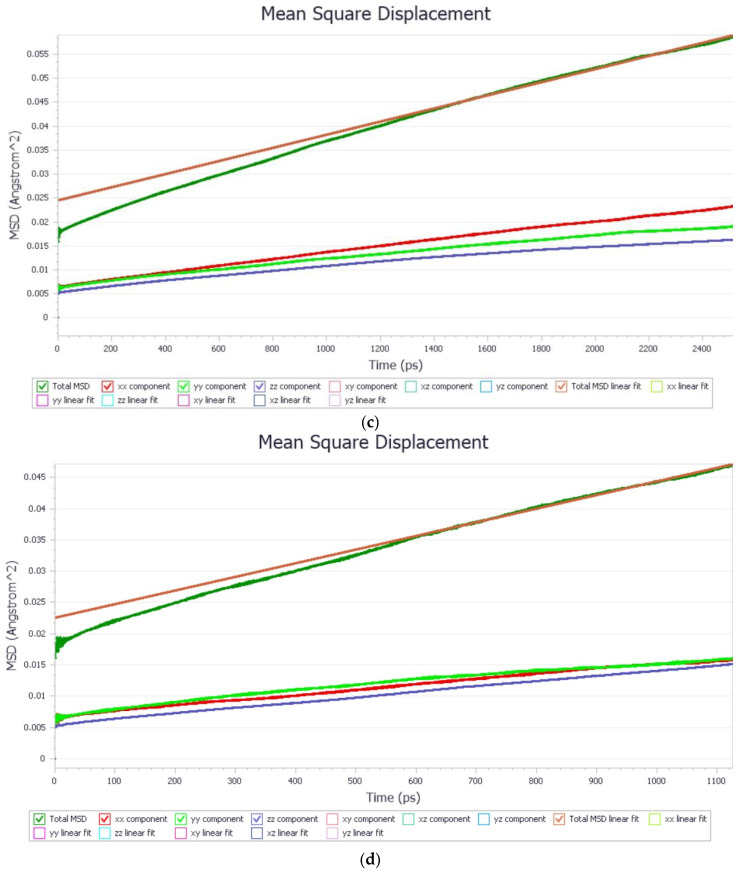
(**a**) Simulation of positive protein fragment in the presence of water molecules with Nb poor (001) (001) surface. The color code for atoms are as follows: grey (carbon), blue (nitrogen), yellow (sulfur), cyan (niobium), pink (lithium), red (oxygen). (**b**) Simulation of the positive fragment in the presence of water molecules with Nb rich LNO (00−1) surface. The color code for atoms are as follows: grey (carbon), blue (nitrogen), yellow (sulfur), cyan (niobium), pink (lithium), red (oxygen). (**c**)-only-XYZ: mean square displacement of the positive fragment in the presence of water with Nb rich LNO (00−1) surface with the view only with checked directions in x, y, z. (**d**)-only-XYZ: mean square displacement of the negative fragment in the presence of water with Nb poor (001) surface with the view only with checked directions in x, y, z.

Simulations were undertaken to evaluate how explicit water molecules interacted with the surface and fragments in order to understand the solution-based experimental results. The reason for undertaking this study with explicit water molecules was to monitor the interactions between the protein fragment and the ceramic surface, which was postulated to enhance in the presence of water molecules. Furthermore, we wanted to compare the effect of Nb rich and Nb poor surfaces, and so simulations were repeated for both surfaces. Results shown in Figure 7a–c and Table 5 indicate that due to the solute-solvent interaction, the protein attachment to the ceramic surfaces varied.

A rule-based UNIVERSAL forcefield was used for the calculation. We used the charges we obtained from DFT simulation based on hirshfeld charges. We decided to look into the same protein fragment on the LNO surfaces, starting with the positive fragment, and to compare fragments with two different surfaces (001) and (00−1). The molecular dynamics (MD) calculations were performed with the LNO surface constrained; the water molecules and the protein fragments moved freely within the periodic boundary conditions. The calculations were carried out for 5 ns, and frames were captured after every 5000 steps. Figure 7a,b are the results obtained after the MD run with the positive protein fragment in water over Nb rich and Nb poor surfaces. The simulation was performed in order to compare with the experimental findings and to determine the role of water on the adsorption of FBG on the LNO surfaces.

**Figure 8:**
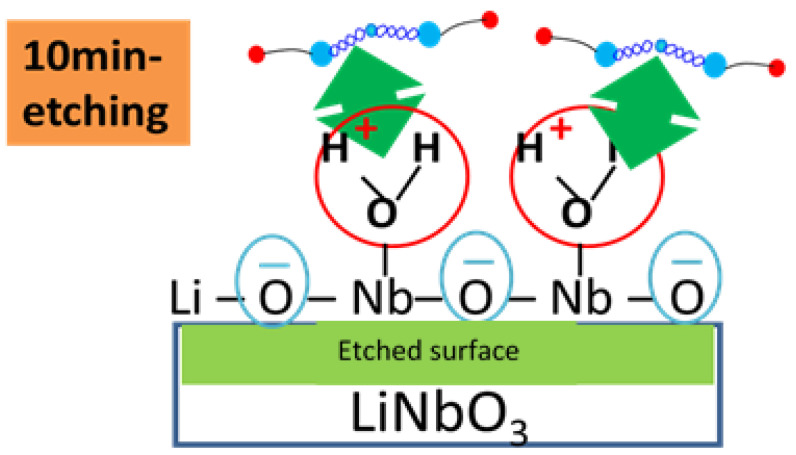
Illustration of proposed binding of FBG binding on a chemical etched LNO surface on the Nb sites (not to scale).

**Figure 9:**
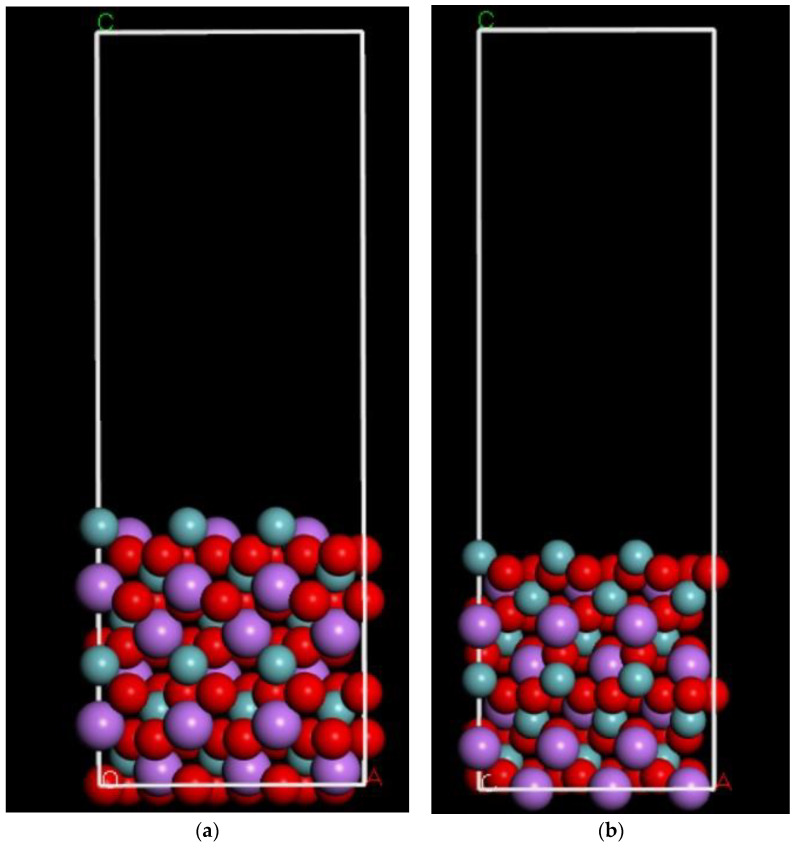
(**a**) Atomistic structure of LNO (001) 3 × 3 × 1 surface model (**b**) LNO (00−1) 3 × 3 × 1 surface model created from LNO bulk structure. The color code for atoms are as follows: cyan (niobium), pink (lithium), and red (oxygen).

The binding energies of the protein fragments on the LNO surfaces were calculated in the presence of water molecules, and the results are shown in Table 5. The energy order remained nearly the same while comparing the binding energy of the protein fragment in the presence and the absence of water molecules. The Nb rich surface showed more favorable binding with water, and hence FBG binding may be enhanced in water as depicted by experiment analysis (Figure 8). The energy difference between that of Nb rich and Nb poor in the presence of water was about 17 kcal/mol, which was more than twice more stable than that in the absence of water molecules. There was no experimental evidence to quantify the above except what is proposed in the image in Figure 9 that the binding of protein was favored by the presence of water, which can now be supported by the binding energy calculation.

Figure 7a,b illustrates the interaction scenario of the etched LNO surface in the presence of water molecules. The reason for the change in the fragment interaction in the presence of water was related to the Nb rich moiety, which enhanced the interaction of water molecules with the surface and favored FBG binding. A similar trend was also observed from the simulation; for the Nb rich surface, water molecules came close to the surface and remained there within the time span of the MD simulation, and the FBG binding with the surface was more favorable. Moreover, we compared the LNO surface of the Nb poor condition, which showed weak water binding ability due to the higher density of oxygen on the surface. In addition, we observed in the simulation that FBG was moving away from the surface and the binding energy was much lower compared to the Nb rich surface. The results were obtained from a classical molecular dynamics approach where we observed that the protein fragment moved away from the surface for the Nb poor surface. This result might be attributed to the hydration of protein moiety with water molecules that corresponded well with the hydration of FBG over mica in the presence of water [31] and verified the fact that FBG becomes hydrated, and that determines its adhesion behavior.

We compared the Mean Squared Displacement (MSD) for the protein fragment in the presence of water molecules for both the Nb rich and poor surfaces. The results are shown in Figure 7c, only-XYZ. The results show the total MSD of the protein fragment with respect to the two surfaces: Nb rich and Nb poor for each of the six Cartesian anisotropic components (that is, xx’, yy’ and zz’,) and their isotopic average. We know this cannot be an absolute proof of the affinity of the protein, but in support of the binding energy, we also wanted to show the change in the diffusion coefficient, with different surfaces with one specific protein fragment for comparison. The results clearly show that the mobility of the protein fragment was different in the presence of water when close to the Nb rich surface compared to the Nb poor surface. The results are shown in Table 6.

This was shown very clearly with the electrostatic potential calculation that the reason for this binding was charge transfer. We see a dramatic electrostatic potential change depending on the concentration of Nb on the surface (Nb rich or Nb poor). The electrostatic potential difference attracts the FBG moiety to bind.

The electrostatic potential calculation from DFT hinted at the dependency of polishing of niobate surfaces. Once we ran the dynamics calculation, we figured out that the nature of the affinity of water with different moieties on the niobate surface is detrimental for the protein affinity. This was compared with the experimental cartoon, as shown in Figure 9, to show that the protein can bind favorably once the niobate surface has more water molecules close to Nb ions on top of the surface.

A recent paper of Cordero-Edwards [32] indicated preferential adsorption on different faces of LNO depending on water pressure. The model proposed is supported by AFM results of FBG binding on graphite surface [33]. The study is well supported by the fact of surface hydroxylation by XPS. We do see a similar correlation in our system as well to validate the understanding. A simple schematic diagram of the binding of the protein with Nb via surface adsorbed water molecules is proposed in Figure 8.

In this paper, we have used a range of molecular simulation techniques from the Monte Carlo method, through Molecular Mechanics and some DFT methods. These multi-scale methods are important to understand interactions of hybrid materials, such as the one we have studied. The calculation procedure was first to identify the best surface in terms of stability, followed by the adsorption energy of the guest over the surface to see the specific configuration. This then should be guided by the DFT calculation to find the electronic structure to know the reason for the binding, which in this case is a change in surface polarity. Then we extrapolated the calculation to a solvated medium to compare the effect in the gas phase and the solvent phase. This approach, therefore, can be expandable to any related material of interest and sets a priori methodology using a unique platform available in the Materials Studio software environment, as provided by BIOVIA Dassault Systemes.

## 3. Experimental Section

### 3.1. Material Preparation and Interfacial Analysis

Polished 10 × 10 × 1 mm (001) stoichiometric (49.9/50.1) z-cut LNO single crystal-single domain substrates were supplied by SWING Ltd., Tsukuba, Japan. Due to a proprietary sample preparation process, each side of the sample had either a positive or negative polarity, hence, single domain. The samples were etched with concentrated HF and HNO_3_ in a 1:2 ratio, both supplied by Wako Chemical Ltd. (Wako, Japan), for 10 min to remove the polishing damaged surface layer prior to any protein adsorption and characterization. A special holder was prepared of silicon rubber to hold the LNO substrate flat in line with a 10 mW He-Ne laser in a LEZA 600 Otsuka Electronics Ltd. (Osaka, Japan) fluid flow cell for zeta potential measurements. We used a 10 mM NaCl solution with 0.1 N HCl and NaOH as the acid and base, respectively, to control the pH and measure the LNO zeta potential between a pH of 2 and 8. Both sides of the LNO substrate were measured, i.e., positive side (001) and negative side (00−1). We carried out an additional measurement of the LNO zeta potential using 0.005 mM and 0.1 mM of LiCl; and 0.05 mM and 0.1 mM solutions of NbCl_5_, respectively.

XPS measurement of the LNO surface before and after FBG adsorption was analyzed using a PHI Micro-XPS with Al X-rays with a beam size of 100 μm, 25 W, and 15 kV, and take-off angle of 45. We collected both wide and narrow scans from 0 to 1200 eV, energy step 1 eV, time/step 20 ms, and narrow scans around C, O, N, Li, and Nb peaks with an energy step of 0.1 eV, time/step of 50 ms.

### 3.2. FBG Adsorption

A 50 mM phosphate-buffered (PB) solution was prepared from NaH_2_PO_4_ and Na_2_HPO_4_ from Wako Chemical Co. Ltd. (Wako, Japan). We adjusted the pH of the solution to 7.0. We used Human FBG from Calbiochem Co. Ltd. (San Diego, CA, USA) to prepare a solution with concentrations from 0.001 mg/mL to 0.5 mg/mL in the PB solution at 37 °C for the XPS surface measurement-based calibration curve. We then applied 5 mL of the solution to the LNO substrate and dried it to prepare a calibration curve of concentration of protein on the LNO substrate surface.

## 4. Molecular Model and Simulation Methods

### 4.1. Molecular Model

#### 4.1.1. LNO Surface Simulation

In order to replicate the experimental conditions under which adsorption took place, it was necessary to prepare the LNO surface structure in a similar manner for simulation. LNO structure was obtained from “The Materials Project” database with symmetry group R-3C and the lattice parameters a = b = 5.26944 and c = 13.9031 and alpha = beta 90.0 and gamma = 120.0. The structure was then cleaved along the (001) Miller plane. We created two surfaces (00−1) and (001) to represent Nb rich and Nb poor surfaces for all the simulations.

Next, we expanded the cleaved crystal into a 3 × 3 × 1 (w × l × h) supercell that would be large enough to accommodate the active FBG regions of interest. The atomic structure of the surface models is shown in Figure 9 respectively. The surface size was expanded to ensure that enough surface area was available for the protein to maneuver, balancing between computational time and accuracy within the periodic boundary condition. We have done a simulation experiment with single-point energy by varying vacuum thickness and slab thickness in terms of the change in energy to get the best value for the same to adopt in the model. We considered a 12 atomic layered LNO (001), (00−1) Å surface for the adsorption energy calculations taking into account the protein fragment size.

A reasonable volume approximately 2.5 times the thickness of the LNO slab combined with the FBG fragment depth along with a 3.5 Å forced gap between the protein fragment and the surface for the vacuum slab was then added on top of the surface plane. We considered this to give the protein fragment the needed space to maneuver within the periodic boundary condition. We optimized the vacuum slab as well to keep the interaction from the top due to the periodic boundary condition to balance the computational effort and accuracy.

Finally, we pasted the protein fragment into the same window as the LNO crystal and positioned it parallel to the surface approximately 3.5 Å apart above and away from it to remove any biases from the initial condition. The Dassault Systèmes BIOVIA Materials Studio 2020 (Vélizy, France) was used to create the respective models.

#### 4.1.2. Protein Model

We obtained the structural data of FBG in the PDB format from the Protein Data Bank ID 3GHG. In addition, the study of fibrinogen molecules using protein modeling may help us understand the causality and effect of novel genetic mutations. This is currently the key method for new mutations in patients with congenital fibrinogen disorders [34].

### 4.2. Simulation Methods

We applied a multi-scale simulation method for the current calculations. The methodology consisted of analyzing the electrostatic potential of the protein fragment, followed by designing the LNO bulk structure and morphology from possible surface elemental arrangements to choose the most stable surface. Once that was known, the protein fragment was modeled based on the electrostatic potential with positive and negative charge centers to calculate the adsorption energy over the LNO surface using the Monte Carlo method. Finally, the conformation used to calculate the local adsorption energy was chosen as the starting configuration for molecular dynamics calculation in the gas phase. The gas-phase calculation was performed in vacuum, and the other calculations were conducted in the presence of water as the solvent phase. Water molecules were packed using Monte Carlo method for the starting configuration of the Molecular Dynamics run. All the calculations presented in the simulation methods section were performed using Dassault Systèmes BIOVIA software programs. We used BIOVIA Materials Studio 2020 to perform the calculations and to generate the graphical results.

#### 4.2.1. Surface Electrostatic Potential Calculation

The reactivity of FBG and other proteins was first investigated through the Surface Electrostatic Potential (SEP) calculation, using the DelPhi [35] method within the Dassault Systèmes BIOVIA Discovery Studio. We obtained the computational results by using the Dassault Systèmes BIOVIA Discovery Studio software program.

DelPhi [35] is a scientific application software, which employs the Poisson–Boltzmann formula on a cubic lattice using the finite-difference technique to calculate the electrostatic charge distribution in and around macromolecules. It incorporates the effects of ionic strength-mediated screening by evaluating the Poisson–Boltzmann equation at a finite number of points within a three-dimensional grid box.

#### 4.2.2. Morphology Method

Morphology of the LNO bulk was calculated using the Bravais–Friedel Donnay–Harker (BFDH) method [36,37,38] as available within the morphology module of Dassault Systèmes BIOVIA. This is a geometrical calculation that uses the crystal lattice and symmetry to generate a list of possible growth faces and their relative growth rates. From this calculation, we deduced the crystal morphology. The BFDH method combines these observations using the Donnay–Harker rules [39] to isolate the likely growth planes, then the Bravais–Friedel rules to deduce their relative growth rates. The method is an approximation and does not account for the energetics of the system. The stronger the bonding effects in the crystal, the less accurate the method becomes. In many cases, however, one can get good approximations, and the method is always useful for identifying important faces in the growth process. The calculations were done with a minimum d (hkl) of 1.3 Å and with a maximum for the (7 Å) plane distance to generate a list of possible growth faces and their relative growth rates.

#### 4.2.3. Adsorption Calculation

We calculated adsorption energies using Monte Carlo [37,38] searches of the configurational space of the substrate–adsorbate system as the temperature was slowly decreased in the Adsorption Locator module in Dassault Systèmes BIOVIA. Adsorption energy calculations were performed using the UNIVERSAL forcefield [40] without water molecules. We used Universal forcefield as it has the full coverage of elements, especially for the metallic elements of Li and Nb. We used the charges calculated using Density Functional Theory (DFT) methods for both the protein fragment and the oxide surface and used that charge while using UNIVERSAL forcefield to improve the accuracy. We described the details of the DFT method in the text. Universal Forcefield is a purely diagonal and harmonic forcefield. Bond stretching is described by a harmonic term, angle bending by a three-term Fourier cosine expansion, and torsions and inversions by cosine-Fourier expansion terms. The van der Waals interactions are described by the Lennard-Jones potential. Electrostatic interactions are described by atomic monopoles and a screened (distance-dependent) Columbic term.

In the Metropolis Monte Carlo method adopted in Adsorption Locator calculation, the ratio of the conformer, rotation, translation was considered with a 32% probability, whereas the ratio of regrowth step was set at around 4% to make the total 100%. We performed the calculations with 50,000 steps, and 5 temperature cycles were chosen for simulated annealing.

#### 4.2.4. Amorphous Packing

We performed the amorphous packing of water molecules within the constructed layer of LNO and protein fragment using a thermodynamic approach [41]. This is available as an Amorphous Cell module in Dassault Systèmes BIOVIA software. In this approach, we packed the molecules in a cell using the well-known Rotational Isomeric State (RIS) model [42] method by minimizing close contacts between atoms whilst ensuring a realistic distribution of torsion angles for any given forcefield. During the packing, the atoms in the framework were fixed. All calculations were performed using the UNIVERSAL forcefield with the charges, as calculated by the DFT method described later in the text. We packed the water molecules with a molar ratio of 1 and targeting a density 3 g/c.c. to fill the surrounding protein and the surface with water molecules. The calculations were performed with Ewald summation for the electrostatic forces and atoms based on the Van Der Waals forces. The temperature set for packing was 298 K. The structures were optimized after construction. This density of the system with LNO and the protein fragment was around 1.5 g/c.c. To pack the vacuum portion well surrounding the protein fragment, a maximum density of 3.0 g/c.c. was chosen, and it was tested to see that no more water molecules could be get packed to that density in order to get the system completely saturated with water. UNIVERSAL may not be the best forcefield to represent water molecules, but the intention was to compare the adsorption of protein fragment over LNO surfaces in the presence and absence of water, so we adopted the same forcefield for the comparison.

#### 4.2.5. Molecular Dynamics Method

The basis of this simulation was the classical equations of motion. Equations were modified, where appropriate, to deal with the effects of temperature and pressure on the system. The general idea is as follows. Given the initial coordinates and velocities and other dynamic information at time *t*, the positions and velocities at time *t* + Δ*t* were calculated. The time step Δ*t* depends on the integration method as well as the system itself [41]. The algorithm is included in Forcite module of Dassault Systèmes BIOVIA. The main output of a dynamics run was a trajectory file that recorded the atomic configuration, atomic velocities, and other information at a sequence of time steps. We analyzed them subsequently. All these calculations used UNIVERSAL forcefield with the charges adopted from the DFT methods described later in the text. We used NHL thermostat [43].

We used Ewald summation for the electrostatic interaction and atom-based summation method for Van Der Waals forces. All the Molecular Dynamics calculations were carried out in two steps. First, a NVT ensemble with a time step of 1 fs and with a total time step of 1 ns was used to equilibrate the system. Once the system was equilibrated, then for the production, we used NVT ensemble with a time step of 1 fs and with a total time step of 5 ns. It may be good to go for a larger time step, but to balance computational time and efficiency, we did not perform longer calculations.

#### 4.2.6. Density Functional Theory Method

In this study, all calculations of the protein fragment, water molecule, and LNO surfaces were performed with DFT [44,45,46] using the DMol3 module of Dassault Systèmes BIOVIA. A gradient corrected functional PBE [31], and DNP basis set was used throughout the calculation. Spin unrestricted calculations were performed using formal spin as initial, which means the initial value for the number of unpaired electrons for each atom was taken from the formal spin introduced for each atom. Basis set superposition error (BSSE) was also calculated for the current basis set in non-local density approximation (NLDA). Geometries were optimized using analytic gradients and an efficient algorithm, which used delocalized internal coordinates so that the change in energy and the change in the maximum force was 2 × 10^−5^ Ha, and 0.004 Ha/ Å, respectively. The maximum displacement considered was 0.005 Å. The SCF tolerance was set to 1 × 10^−^^5^. Direct inversion of the iterative subspace (or DIIS) technique developed by Pulay [47] was used. Charge calculations were performed, and the Hirshfeld [20] charges were calculated for water, protein fragment, and the LNO surfaces.

## 5. Conclusions

In the current study, we compared experimental observations of preferential binding of FBG over LNO both in the gas phase and solvent phase to examine the dependency of surface polishing. Experimentally, it was observed that the FBG binding was subtly favored in the presence of water molecules, and the interaction was pronounced with Nb rich LNO surface, which happens only after polishing. The adsorption energy of the proteins in the gas phase showed that the energy range was almost similar, as observed in the experimental zeta potential results. This needs to be explored more carefully by experiment, as LNO surface polarization is key for its different applications, including redox reactions as applications to fuel cells. A multi-scale simulation method was adopted to find the binding probability of the protein fragment with nucleophilic and electrophilic sites interacting with Nb rich and poor surface, which were identified as morphologically independent surfaces (001) and (00−1). The surface interaction was favored when the surface was Nb rich due to the high electrostatic potential, which was compensated by the water molecules coming close to the surface and then binding with the protein. The multi-scale simulation has clearly shown the difference between the gas phase and solvent phase binding, as well as the effect of polishing. This reiterated the fact, which is observed for this type of oxide, that surface polishing changes the charge on the surface, and its binding to protein is favorable in the presence of water. This is the first simulation to our knowledge to include both experimental observations, where a protein fragment binds with a piezoelectric surface in the presence of water molecules, and a simulation. These results may lay the foundation to formulate a priori rule related to how surface polarity can be used to design biosensors for protein adsorption with active ceramic surfaces.

## Figures and Tables

**Table 1:** Correlation of Surface area and miller planes of possible growth surfaces in LNO (correction at the h k l value).

h k l	Multiplicity	D _hkl_ (Å)	% Total Facet Area
0 0 1	1	2.3105	49.5433
0 0 −1	1	2.3105	49.5433
−1 1 −2	3	3.7498	0
1 −1 2	3	3.7498	0
−3 2 1	6	1.6728	0.456671
−3 2 2	6	1.6374	0.456671

**Table 2:** Adsorption energy for (001) LNO surface and protein fragments in a vacuum. The number of atoms in the parenthesis represents the number of atoms present in the protein fragment to calculate the average adsorption energy.

Fragment Nature	Adsorption Energies (kcal/mol)	Average Adsorption Energy/Per Atom for Protein Fragment (kcal/mol)
Positive	−906.3	−4.89 (185)
Negative	−718.1	−4.10 (175)

**Table 3:** Adsorption energy for (00−1) LNO surface and protein fragments in a vacuum. The number of atoms in the parenthesis for average adsorption energy represents the number of atoms present in protein fragment.

Fragment Nature	Adsorption Energies (kcal/mol)	Average Adsorption Energy/Per Atom of Protein Fragment (kcal/mol)
Positive	−713.2	−3.85 (185)
Negative	−856.1	−4.89 (175)

**Table 4:** Binding energy of protein fragment with LNO surfaces.

System	Total Energy (kcal/mol)	Average Binding Energy of Protein Fragment ΔE (kcal/mol)
Blank (001) surface	−13,428.29	
Blank (00−1) surface	−13,425.67	
Positive Protein Fragment	−10,651.34	
Negative Protein fragment	−10,548.28	
Positive fragment + (001)	−24,085.78	−6.15
Negative fragment + (001)	−23,980.18	−3.61
Positive fragment + (00−1)	−24,080.34	−3.33
Negative fragment + (00−1)	−23,978.23	−4.28

**Table 5:** Binding energies of protein fragments on LNO surfaces in the presence of water.

System	Total Energy (kcal/mol)	Average Binding Energy of Protein Fragment ΔE (kcal/mol)
Blank (001) surface	−13,428.29	
Blank (00−1) surface	−13,425.67	
Positive Protein Fragment	−10,651.34	
Negative Protein fragment	−10,548.28	
1701 Water Molecules	−85,896.09	
Positive fragment + (001)	−109,993.21	−17.49
Negative fragment + (001)	−109,879.38	−6.72
Positive fragment + (00−1)	−109,980.21	−7.11
Negative fragment + (00−1)	−109,878.12	−8.08

**Table 6:** Diffusion coefficient from the best fit mean square displacement for protein fragment with Nb rich (00−1) and Nb poor (001) surface in water.

System	Diffusion Coefficient A^2/ps	R^2
Protein Fragment with Nb rich surface (00−1)	3.634 × 10^−6^	0.9972
Protein Fragment with Nb poor surface (001)	2.284 × 10^−6^	0.9965

## Data Availability

The data that support the findings of this study are available from the corresponding author upon reasonable request.
